# ORM 1 as a biomarker of increased vascular invasion and decreased sorafenib sensitivity in hepatocellular carcinoma

**DOI:** 10.17305/bjbms.2022.7268

**Published:** 2022-06-29

**Authors:** Jiangning Gu, Shiqi Xu, Xiang Chen, Haifeng Luo, Guang Tan, Wenjing Qi, Feng Ling, Chenqi Wang, Feiliyan Maimaiti, Yunlong Chen, Lili Yang, Menghong Yin, Dan Chen

**Affiliations:** 1 Department of Hepatobiliary Surgery, The First Affiliated Hospital of Dalian Medical University, Dalian, Liaoning Province, China; 2 Department of Pathology, The First affiliated Hospital of Dalian Medical University, Dalian, Liaoning Province, China; 3 Department of Sports Medicine, Dalian Municipal Central Hospital, Dalian Medical University, Dalian, Liaoning Province, China; 4 Department of Nursing, The First affiliated Hospital of Dalian Medical University, Dalian, China

**Keywords:** Orosomucoid 1, microvascular invasion, sorafenib sensitivity, hepatocellular carcinoma

## Abstract

This study aimed to clarify the role of Orosomucoid 1 (ORM1) in the development and therapy resistance in hepatocellular carcinoma (HCC). The mRNA expression level of ORM1 was analyzed through integrative analysis of Gene Express Omnibus and The Cancer Genome Atlas datasets. The protein expression level of ORM1 in our cohort was determined by immunohistochemistry. Correlation analysis was used to investigate the relationship between ORM1 expression and clinical parameters. The cell counting Kit-8 assay was used to clarify the role of ORM1 in HCC malignant behaviors, including cell growth and sorafenib sensitivity, *in vitro*. The results indicated that ORM1 was significantly downregulated in the hepatic cancer cells compared with the levels in non-cancerous cells; however, it was upregulated in microvascular invasion samples, especially in the cancer embolus, compared with that in the surrounding tumor cells. Although Kaplan–Meier analysis did not show an association between ORM1 expression and the overall survival rates of patients with HCC, univariate analysis indicated that ORM1 expression was highly correlated with tumor grade and stage. An *in vitro* assay also revealed that downregulation of ORM1 led to the suppression of tumor growth and enhancement of sorafenib sensitivity without epithelial-to-mesenchymal transition alteration, which was consistent with our bioinformatic analysis. Hence, ORM1 plays a key role in HCC tumorigenesis and may serve as a potential target for the development of therapeutics against HCC.

## INTRODUCTION

Hepatocellular carcinoma (HCC) is one of the leading causes of cancer-related deaths worldwide. According to 2019 cancer statistics, the five-year survival rate of HCC remains approximately 15%, making it one of the most malignant cancers [1], largely due to late diagnosis, low response to chemotherapy, and high probability of recurrence. The situation is even more serious in China, as many hepatitis B or C patients may develop HCC, resulting in cirrhosis and further tumorigenesis [2,3].

Although the association between chronic inflammation and the development of hepatic cancer is well-documented, the specific mechanisms underlying this association remain largely unknown [4,5]. However, it is widely accepted that long-term inflammatory stimuli result in the activation of malignant transformation. Thus, signaling pathways associated with inflammation-related stress may be involved in tumorigenesis [6,7]. Orosomucoid 1 (ORM1) is one of the most well-characterized acute phase proteins that have been associated with many cellular pathways, including immunity, tissue regeneration, metabolism, and cancer development which may help tumor cells prevent cellular decay as a result of the adverse environment [8,9]. The expression of ORM1 is also altered in response to various health conditions, such as chronic inflammation, infection, injury, drug sensitivity, and cancer [10-12]. However, the expression levels of ORM1 vary among different cancers. Jia et al. studied ORM1 expression in various tumors and their adjacent normal tissues and found that ORM1 expression decreased in liver cancer, cholangiocarcinoma, esophageal carcinoma, and lung squamous cell carcinoma. whereas it increased in invasive breast carcinoma and colon adenocarcinoma, among others[13]. However, its role in HCC remains largely unknown. Theoretically, viral hepatitis is an example of a chronic stimulus that may alter ORM1 expression and thus play an important role in the transition from chronic hepatitis to hepatic cancer. ORM1 is mainly synthesized in the liver and acts as a typical acute phase protein [10]; therefore, we hypothesized that ORM1 expression may be related to tumorigenesis and drug effects in HCC.

In this study, we found that ORM1 is differentially expressed between tumor and non-tumor tissues and was associated with tumor growth, drug sensitivity, and microvascular invasion (MVI). Thus, ORM1 could be regarded as a potential target for the development of future therapeutics against HCC.

## MATERIALS AND METHODS

### Gene expression datasets

Gene expression datasets (GSE10143, GSE45114, GSE45267, GSE75620, and GSE93595) were downloaded from the GEO database. GSE10143 included 80 tumor and 82 non-tumor tissues, and GSE45114 included 24 tumor and 25 non-tumor tissues. Of the 24 HCC tissues, 21 were without MVI and three were pathologically confirmed to have MVI. GSE45267 contained 48 HCC and 39 non-cancerous tissues. The GSE93595 dataset is the RNA-seq data of Huh-7 naïve and sorafenib-resistant cells from xenografts, while GSE75620 is the RNA-seq data of Huh-7 naïve cells treated for a short period with sorafenib. The Cancer Genome Atlas (TCGA) datasets were used for correlation and survival analyses of the selected candidates.

### Patients and tissues collection

15 pairs of HCC samples with adjacent non-tumor tissues and 5 HCC samples with MVI were collected from patients who underwent surgical resection at Department of Hepatobiliary Surgery of the first affiliated hospital of Dalian Medical University. All specimens were confirmed by senior pathologists, and none of the patients received antitumor treatment before surgery. Clinicopathologic data are listed in Table S1.

### Selection of differentially expressed genes

DESeq2 [14] in R statistical software (version 3.6.2) was used to identify differentially expressed genes (DEGs) from the different GEO datasets. Student’s *t*-test was used to evaluate statistical significance, and the threshold was set to *p* < 0.05, with a fold change ≥2. Probes without a corresponding gene symbol were removed, and volcano and box plots produced using ggplot in R were used to visualize the DEG data.

### Functional enrichment analysis

**FIGURE 1:**
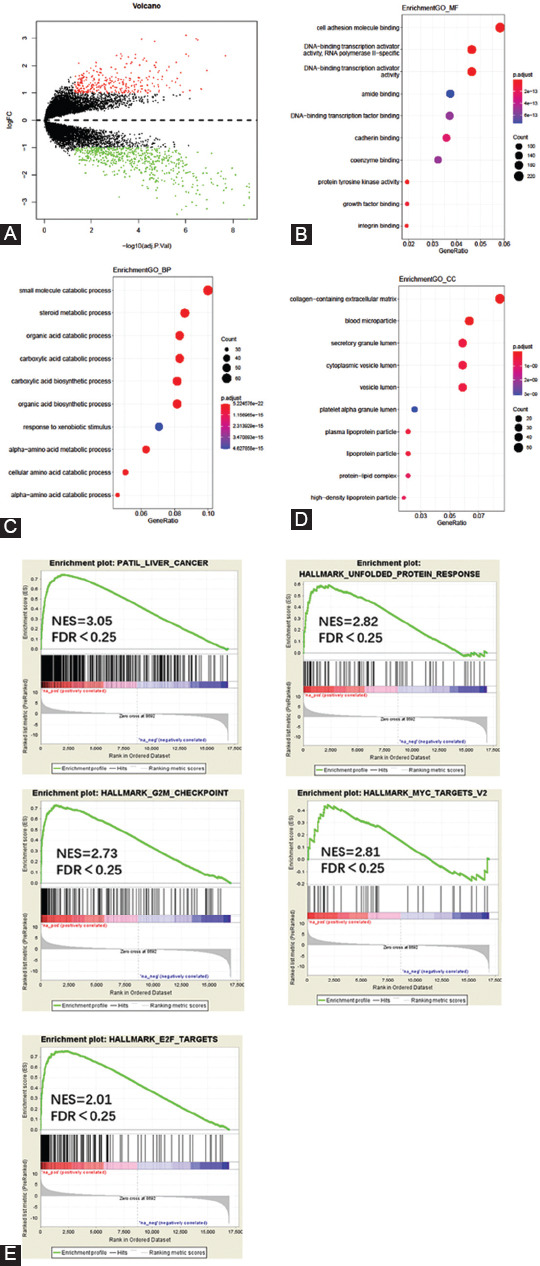
Selection of differentially expressed genes (DEGs) between hepatocellular carcinoma and non-tumor tissues. Volcano plot shows the DEGs isolated from the GSE45114 dataset (A); Red dots represent upregulated genes, and green dots represent downregulated genes; GO term enrichment analysis results for the DEGs (B-D); GSEA analysis of the GSE45114 dataset (E)

Gene ontology (GO) and gene set enrichment analysis (GSEA) were performed to evaluate the functional enrichment of candidate genes. The GO results were ranked by enrichment score; the top 10 terms are listed in Figure 1. GSEA was conducted using software developed by the Massachusetts Institute of Technology and Harvard University [15]. Associations with *p* <0.05 and FDR <0.25 were considered relevant.

### Survival analysis

Kaplan–Meier analysis based on the clinical information in the GEO and TCGA databases was performed using R statistical software (version 3.6.2). The log-rank test was used to identify significant differences between the two groups, and *p* < 0.05 was considered significant.

### Cell culture

Huh-7 and HepG-2 cell lines were purchased from the American Type Culture Collection (Manassas, VA, USA) and authenticated by DNA typing at the Shanghai Jiao Tong University Analysis Core. The cells were cultured in Dulbecco’s modified Eagle’s medium (Gibco, Thermo Fisher Scientific, Waltham, MA, USA) supplemented with 10% fetal bovine serum (Gibco), 0.1 mM sodium pyruvate (Gibco), 50 U/mL penicillin (Gibco), and 50 mg/mL streptomycin (Gibco) at 37°C under 5% CO_2_ in a humidified chamber.

### Quantitative reverse transcription-polymerase chain reaction

Total RNA was isolated using a Qiagen RNeasy Plus Mini Kit according to manufacturer’s protocol (Cat. No. 74134; Qiagen, Hilden, Germany). RNA quality and concentration were evaluated using a Nanodrop 2500 spectrophotometer (Thermo Fisher Scientific). One microgram of RNA was used to synthesize first-strand DNA (#R211-02; Vazyme, Nanjing, China), and quantitative reverse transcription-polymerase chain reaction (qRT-PCR) was performed using the SYBR Green PCR method on a Roche LightCycler 480 (Roche Life Science, Penzburg, Germany). The 2-ΔΔCt method was used to analyze the relative expression of ORM1. The primer sequences used for ORM1 were 5′-ACACCACCTACCTGAATGTCC-3′ (forward) and 5′-GTGAGCGAAATGCTCTTGGC-3′ (reverse). CDH1:5′- CGAGAGCTACACGTTCACGG-3′ (forward) and 5′-GGGTGTCGAGGGAAAAATAGG-3′ (reverse). CDH2: 5′- TGCGGTACAGTGTAACTGGG-3′ (forward) and 5′- GAAACCGGGCTATCTGCTCG-3′ (reverse). ZEB1: 5′- TTACACCTTTGCATACAGAACCC-3′ (forward) and 5′- TTTACGATTACACCCAGACTGC-3′ (reverse). BCL-2: 5′-GGTGGGGTCATGTGTGTGG-3′ (forward) and 5′ -CGGTTCAGGTACTCAGTCATCC -3′ (reverse). CASP3: 5′- AGAGGGGATCGTTGTAGAAGTC-3′ (forward) and 5′ - ACAGTCCAGTTCTGTACCACG-3′ (reverse). Human β-actin was used as an internal control. The siRNAs targeting ORM1 were synthesized by Sangon Biotech. The sequences were as follows: si-ORM1-1, 5′ -CUAUAACACCACCUACCUGAATT-3′ (sense) and 5′ -UUCAGGUAGGUGGUGUUAUAGTT-3′ (antisense). Si-ORM1: CAGAUGUCGUGUACACCGAUUTT-3′ (sense) and 5′ -AAUCGGUGUACACGACAUCUGTT-3′ (antisense). The scrambled sequence was used as the control.

### Cell growth assay

Cell proliferation was evaluated using the cell counting Kit-8 (CCK-8) assay (TransGen Biotech, Beijing, China). HepG-2 and Huh-7 cells were seeded in 96-well plates at densities of 1 × 10^3^ and 3 × 10^3^ cells/well, respectively, and cultured overnight. The number of viable cells was quantified every 24 h for 5 days by measuring the optical density at 450 nm using a microplate reader (Epoch, BioTek, USA).

### Determination of the half maximal inhibitory concentration

The half-maximal inhibitory concentration (IC_50_) value was determined using a standard protocol from our laboratory [16]. Briefly cell seeding was optimized before the initiation of formal experiments to ensure that the cell density was not more than 90% after 72 h of culture. The cells were plated in 96-well plates, and sorafenib (APExBIO, Houston, TX, USA) was added the following day. The highest concentration of sorafenib was 20 μM, and the half-dilution method was used. The CCK-8 assay was used to detect cell viability, and dimethyl sulfoxide (DMSO) was used as the vehicle.

### Immunohistochemistry

Immunohistochemistry (IHC) was used to evaluate ORM1 expression in HCC and adjacent non-tumor samples according to our standard protocol [16]. Briefly, the tissue sections were incubated with rabbit anti-ORM1 antibody (BBI, D221584; Sangon Biotech, Shanghai, China) at 1:100 for 1 h at room temperature. Tissue sections were then incubated with horseradish peroxidase-conjugated secondary antibody (KIHC-1; Proteintech, Wuhan, China) at room temperature for 20 min. Isotype was used as the control. All slides were reviewed and scored by two pathologists who were blinded to clinicopathological information. The final scores were calculated as follows: ten 400× magnification fields were chosen randomly, and the staining intensity was scored as 0, 1, 2, and 3 for negative, weak, intermediate, and strong staining, respectively. Then, in each field, the percentage of positive cells was scored as follows: 0 (<5%), 1 (5–25%), 2 (26–50%), 3 (51–75%), and 4 (>75%). The IHC score, calculated by adding the two scores, ranged from 0 to 12. This study was approved by the Ethics Committee of the First Affiliated Hospital of Dalian Medical University.

### Ethics approval and consent to participate

This study was approved by the Ethics Committee of the First Affiliated Hospital of Dalian Medical University of Science and Technology. The approval number is PJ-KS-KY-2022-110. All patients provided written informed consent, which was obtained from the study participants before study commencement.

## RESULTS

### DEGs in HCC and non-tumor tissues

We first identified the DEGs in HCC and adjacent non-tumor tissues using the GEO datasets. In total, 919 DEGs were identified from GSE45114, 354 of which were upregulated, and the remaining were downregulated (Figure 1A). Functional analysis using GO analysis indicated that these DEGs were associated with immune response, cell adhesion, and cell metabolism, which are listed in Figure 1B-D. GSEA showed that the DEGs were also highly correlated with cellular metabolism, the PI3K-AKT pathway, and E2F targets, all of which are known to be associated with carcinogenesis (Figure 1E).

### ORM1 was downregulated in HCC compared to non-cancerous cells

**FIGURE 2:**
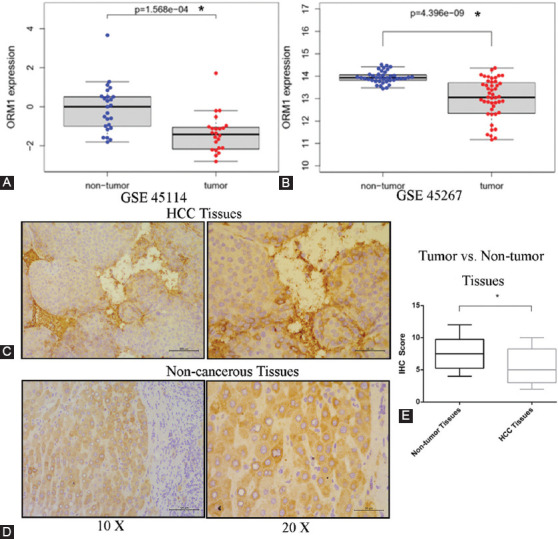
Comparison of ORM1 expression in hepatocellular carcinoma (HCC) tissues and the adjacent non-tumor tissues. (A and B) Differential expression of ORM1 in public datasets GSE45114 (A) and GSE45267 (B). Representative images of immunohistochemistry results for ORM1 expression in HCC tissues and adjacent non-tumor tissues in our cohort (C and D). ORM1 immunohistochemical staining scores for HCC tissues and adjacent non-tumor tissues in our cohort (E)

The expression of ORM1, an acute-phase protein, was significantly downregulated in HCC tissues in both GSE45114 and GSE45267 compared to that of adjacent non-tumor tissues (Figure 2A and B). IHC was used to validate this observation in our own cohort, which contained 15 HCC pathologically confirmed HCC patients (case 1 to 15 in Table S1) with adjacent non-tumor tissues at the protein level (Figure 2C-E). The results indicated that ORM1 was downregulated in hepatic cancer cells compared to that in non-cancerous cells, although it seems that ORM1 had a higher level in the stroma of HCC tissues.

### ORM1 plays a role in MVI in HCC

**FIGURE 3:**
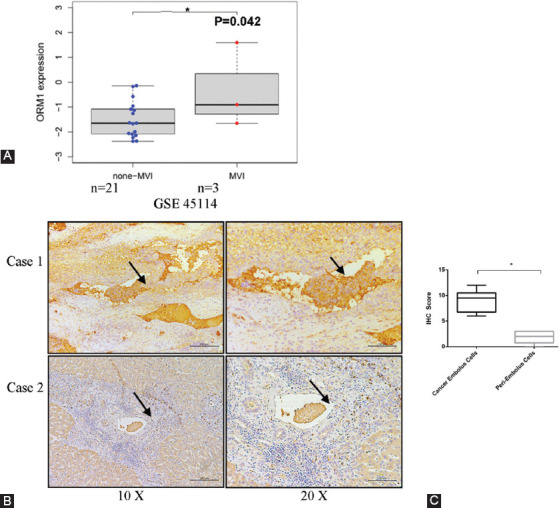
High ORM1 expression is associated with microvascular invasion in hepatocellular carcinoma (HCC). Differential expression of ORM1 in HCC samples with and without MVI from the public dataset GSE45114 (A). Representative image of the immunohistochemistry analysis of cancer cells in microvessels (B). The arrow indicates the cancer cells. Immunohistochemical staining scores for microvascular-invaded cells and the surrounding tumor cells (C)

The previous studies have shown that ORM1 facilitates tumor necrosis factor (TNF)-induced angiogenesis [10,17]; therefore, we hypothesized that this may be correlated with vascular invasion. Accordingly, we analyzed the GSE45114 dataset, which contained HCC samples with and without MVI (*n*=3 and 21, respectively). The results validated our hypothesis that ORM1 was significantly upregulated in the MVI samples (Figure 3A). Simultaneously, IHC was performed in five clinical HCC samples (case 16–20 in Table S1) with pathologically confirmed MVI in our cohort. The results of these assays were in accordance with our bioinformatics analysis, which suggested that ORM1 expression was upregulated in the cancer embolus compared to that in the surrounding tumor cells (Figure 3B and C). Although ORM1 expression was lower in hepatic cancer cells than in non-cancerous ones, vascular invasive tumor cells had higher levels of ORM1.

### ORM1 expression correlates strongly with tumor stage and grade in HCC patients

**FIGURE 4:**
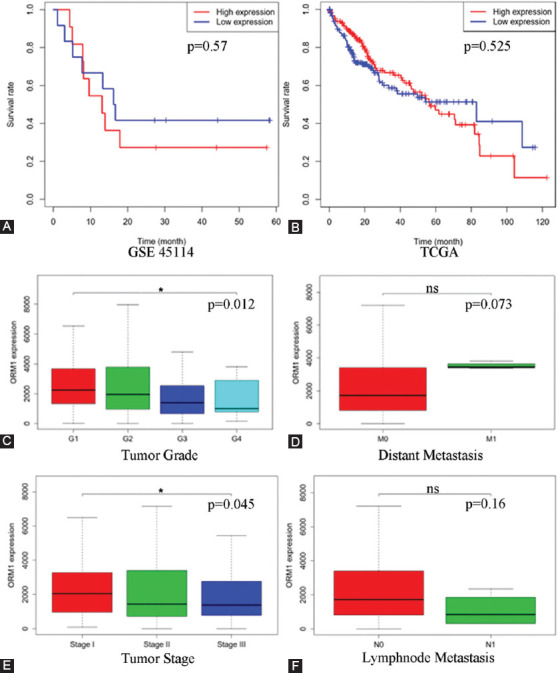
Clinical association of ORM1 expression data from public databases. Kaplan–Meier analysis using a log-rank test for overall survival rates and ORM1 expression in the GSE45114 cohort (A) and TCGA cohort (B). Correlation analysis of ORM1 expression with tumor grade (C), distant metastasis (D), tumor stage (E), and lymph node metastasis (F)

Since ORM1 has already been validated as a marker of cancer transmission and MVI, we hypothesized that it is also a marker of overall survival. ORM1 expression levels did not correlate with overall survival (Figure 4A and B), or distant metastasis, or lymphatic metastasis. However, they were strongly correlated with the tumor stage and grade of the HCC samples from the TCGA database (Figure 4C-F).

### ORM1 expression is associated with sorafenib resistance in HCC

**FIGURE 5:**
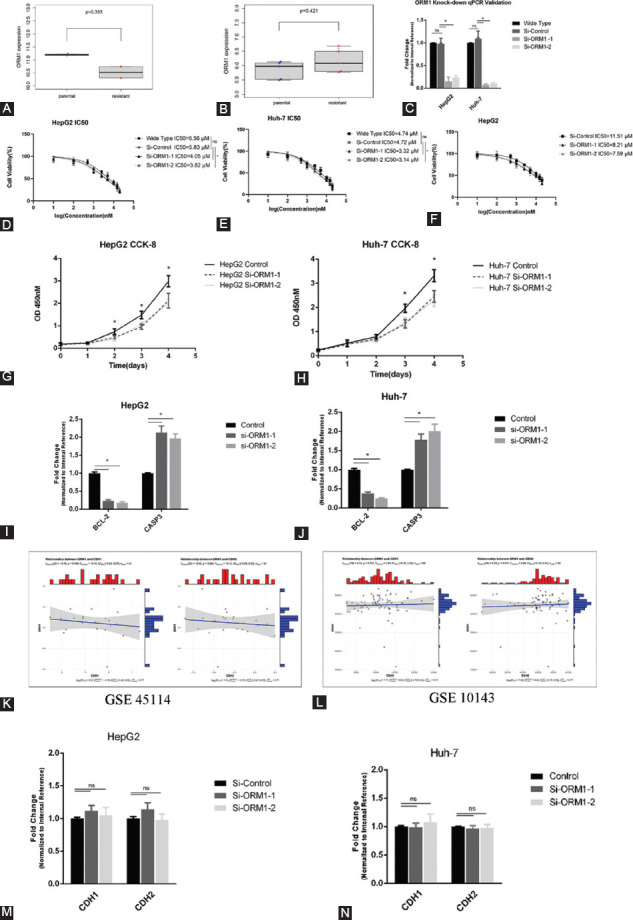
ORM1 expression correlates with cell growth and sorafenib resistance. Differential ORM1 expression in the GSE93595 and GSE75620 datasets (A and B). Knockdown efficiency of two siRNA targeting ORM1 in Huh-7 and HepG2 cells (C). IC_50_ value of sorafenib after ORM1 knockdown in HepG-2 and Huh-7 cells (D and E). IC_50_ value of sorafenib after ORM1 knockdown in long-term sorafenib treatment HepG2 cells (F). Cell growth inhibition after ORM1 knockdown in Huh-7 and HepG-2 cells (G and H). The expression of BCL-2 and CASP3 before and after ORM1 knockdown in HepG-2 and Huh-7 cells (I and J). Pearson correlation analysis of ORM1 with CDH1 and CDH2 in GSE45114 and GSE10413 (K and L). qPCR validation of CDH1 and CDH 2 in HepG2 and Huh-7 cells with ORM1 knockdown by siRNA (M and N)

The previous studies have shown that ORM1 expression serves as a predictor of therapeutic effects in ovarian cancer and lymphoma. Therefore, we investigated whether ORM1 expression induced sorafenib resistance in HCC. Two GEO datasets were used to clarify the relationship between ORM1 expression and sorafenib, using data from resistant and naïve cells (Figure 5A and B). Although the results of GSE93595 seem to indicate that ORM1 expression was lower in resistant samples, there was no significant difference (*p* = 0.333). Furthermore, the results were obtained from the RNA-seq of xenografts instead of cells themselves, and the sample size was small. The results of GSE75620 indicated that ORM1 expression was higher in sorafenib-treated cells; however, there was no significant difference (*p* = 0.421) between cells that underwent short-term treatment with sorafenib and cells with long-term resistant cells. Thus, we investigated whether ORM1 plays a role in sorafenib resistance *in vitro* using our own system. We constructed ORM1-conditioned knockdown cells using specific small interfering RNA in Huh-7 and HepG-2 cells (Figure 5C). The IC_50_ values for sorafenib decreased significantly after ORM1 knockdown in HepG2 and Huh-7 cell lines, suggesting a relationship between ORM1 expression and sensitivity to treatment (Figure 5D and E). Furthermore, we established a HepG2 cell line with 1μM sorafenib long-term treatment, and we detected the IC_50_ of sorafenib before and after ORM 1 knockdown in that cell line (Figure 5F). These results revealed that ORM1 expression was highly correlated with sorafenib sensitivity. In addition, the CCK-8 assay confirmed that ORM1 knockdown attenuated cell growth (Figure 5G and H). We further detected BCL-2 and CASP3 before and after ORM1 knockdown, the anti-apoptotic gene BCL-2 was downregulated whereas the apoptotic gene CASP3 was upregulated after ORM1 knockdown by siRNA (Figure 5I and J). These results were accordance with the CCK-8 assay, which revealed that ORM1 could promote tumor growth, and its knock-down could suppress cancer cell growth. As ORM1 is related to cell growth, microvascular metastasis, and drug resistance, we explored whether it is associated with epithelial-to-mesenchymal transition (EMT), one of the most common processes in cancer. The correlation analysis between ORM1 and CDH1 (epithelial marker) and CDH2 (mesenchymal marker) in public datasets (GSE 45114 and 10413) was not significantly different (Figure 5K and L). The levels of CDH1 and CDH2 were not altered after ORM1 knockdown in HepG-2 and Huh-7 cells (Figure 5M and N). The above results indicated that ORM1 promotes cell growth and drug resistance, but does not influence EMT.

## DISCUSSION

Although great progress has been made in cancer treatment, HCC remains one of the most common malignant tumors worldwide, with poor overall survival and a lack of effective treatment. In China, most patients with hepatitis B virus or hepatitis C virus infection develop HCC [18], because the long-term injury regeneration process facilitated by hepatitis B or C inevitably leads to cell stress and associated consequences, such as tumor development. Thus, investigation of acute phase and stress-related proteins may help to uncover novel HCC treatment targets.

Using a combination of *in vitro*, *in silico*, and immunohistochemistry in clinical samples, we were able to show that ORM1 was differentially expressed in HCC tumor and non-tumor tissues, which was in accordance with the findings of previous studies. Furthermore, in vascular invasion specimens, ORM1 expression was higher in the cancer embolus than in the surrounding tumor cells, suggesting that ORM1 expression may be related to vascular invasion, thereby reducing the overall prognosis of HCC patients. Based on these results, we hypothesized that ORM1 may be correlated with drug sensitivity, as all anti-cancer inhibitors induce cell stress, which must be overcome to facilitate chemoresistance. Although there was no statistical difference, the trend showed that ORM1 expression was higher in the data from sorafenib-resistant cells available in a public database. Therefore, we further performed molecular experiments to validate this upregulation in sorafenib-resistant HCC cell lines. Moreover,ORM1 knockdown was shown to suppress cell growth and restore sorafenib sensitivity to some extent. These results indicate that despite its tumorigenicity, ORM1 may be a useful biomarker for predicting MVI and drug sensitivity.

In our study, ORM1 was downregulated in cancer tissues compared to adjacent normal tissues, but upregulated in cancer embolus. Although seemed paradoxically, many genes have been validated to play dual role in biological process, especially in cancer. Sun et al. reported that FBXO22 was upregulated in breast cancer and promoted cell proliferation and colony formation but suppresses invasion and epithelial-to-mesenchymal transition [19]. In pancreatic cancer cells, downregulation of Ski could decrease tumor growth and promoted cell invasion by regulation the TGF-β signaling pathway [20]. TGFBR3, SnoN, and GRHL2 have also been shown to play dual roles in different types of cancer [21-23]. Similarly, based on our results, ORM1 was validated to be acting as both a tumor suppressor and a tumor promoter in HCC.

ORM1 can also be detected in plasma and other body fluids, making it an ideal candidate for the dynamic monitoring of disease progression. Zhang et al. indicated that higher serum ORM1 expression predicted inferior survival and lower response to chemotherapy in natural killer/T cell lymphoma [17]. In addition, Sun et al. reported that serum ORM1 expression was decreased in non-small cell lung cancer patients, which was in accordance with the results of a study by Jia et al. [13], supporting the application of ORM1 as a biomarker [24]. Other studies have also noted the prognostic and predictive value of ORM1 in cancer diagnosis and treatment for several other cancers, including ovarian and bladder cancers [25,26]. As ORM1 is predominantly synthesized in the liver, hepatic diseases may have more influence on its expression. Kojima et al. reported that ORM1 expression increased in patients who underwent hepatic resection, while mechanistic studies validated the relationship between ORM1 expression and interleukin 6- and TNF-dependent pathways both *in vitro* and *in vivo* [27]. Nonetheless, a consensus has not been reached on whether ORM1 expression is up- or down-regulated in liver cancer compared to non-tumor tissues with different ORM1 expression profiles reported in the previous studies [8,13,28]. This may be partially attributed to the control group because ORM1 is differentially expressed in acute hepatitis, liver cirrhosis, liver failure, and liver cancer [8]. Cancer progression may influence these various results. In addition, except for cancer cells, ORM1 was rich in stroma, which was also seen in our IHC results; thus, it may lead to different results in different studies. In our study, ORM1 was downregulated in cancer cells but upregulated in metastatic sites, with its primary expression focused on the cancer embolus. Moreover, Sun et al. showed that ORM1 expression is downregulated in Stage I NSCLC patients compared to healthy controls but is upregulated in Stage IV patients [24]. This indicates that ORM1 expression changes under different conditions, making it a dynamic measure of disease progression.

Although this study provides valuable insights into ORM1 expression in HCC, it has some limitations. Although we validated the finding that ORM1 expression is downregulated in cancer cells and correlates with vascular invasion and drug sensitivity, we could not validate its negative influence on overall survival. Our external validation using TCGA cohort showed no correlation between ORM1 expression and overall survival. This may be partially attributed to the sample size and the fact that we did not perform an expression pattern analysis in our cohort. Although ORM1 promoted cell growth and drug resistance, it did not alter the EMT process, which means that other mechanisms are involved, and this needs to be further explored. In addition, all experiments were performed *in vitro*, indicating that the exact role of ORM1 in HCC *in vivo* still needs to be evaluated. These studies are currently underway and should provide more insights into the application of ORM1 as a biomarker for disease prognosis.

## CONCLUSION

Although ORM1 was downregulated in HCC tissues, it was positively correlated with microvascular invasion and promoted tumor growth and drug resistance. Moreover, ORM1 plays an important role in tumorigenesis and vascular invasion in HCC, and is a reliable marker of sorafenib resistance. Thus, ORM1 could be regarded as a promising target for HCC therapy in the future.

## Figures and Tables

**Table S1:**
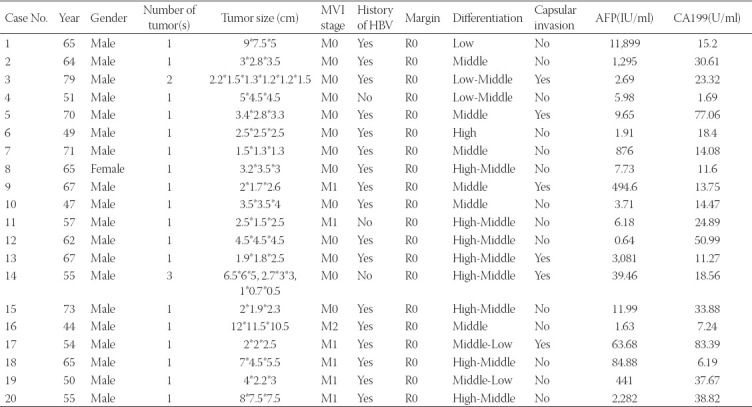
Clinicopathologic data
